# Log-Normal ELISPOT spot size distribution permits count harmonization among different laboratories

**DOI:** 10.1186/2051-1426-2-S3-P156

**Published:** 2014-11-06

**Authors:** Srividya Sundararaman, Alexey Karulin, Nadine BenHamouda, Judith Gottwein, Sreenevas Laxmanan, Steven Levine, John Loffredo, Stephanie McArdle, Christine Neudoerfl, Diana Roen, Karina Silina, Mackenzie Welch, Paul V Lehmann

**Affiliations:** 1 Cellular Technology Ltd., Shaker Hts., USA; 2 Hospital Europeéen Georges Pompidou, France, Paris, France; 3 University of Copenhagen, Denmark; 4 Biogen Idec, Cambridge, MA, USA; 5 Bristol Myers Squibb, New York, NY, USA; 6 Nottingham Trent University, Nottingham, UK; 7 Medizinische Hochschule Hannover, Germany; 8 Pharmasan Labs Inc., Minneapolis, MN, USA; 9 Biomedical Research Study Center, Latvia

## Introduction

ELISPOT assays are primarily used to detect the number of T cells that respond to a given antigen. With that number being absolute for any given donor sample, ELISPOT counts should be similar between laboratories, if subjectivity in counting is avoided. Due to the differences in spot sizes ranging from microns to millimeters, setting cut offs for minimal and maximal spot sizes will lead to substantial variability between investigators when determined subjectively, irrespective of experience. In contrast, if spot size distributions would follow predictable statistical functions, objective gating decisions could be made using common standards, eliminating subjective calls of the counting process. This study aims to determine if ELISPOT size distribution follows predictable statistical distributions and therefore if ELISPOT counting can be made objective based on statistical principles.

## Methods

In order to study whether spot sizes follow predictable functions, we studied the size distributions of ELISPOT assay results obtained with 24 donors and 32 individual viral peptides of Cytomegalovirus, Epstein Barr, and Influenza virus activating CD8 cells, and the CMV and EBV virions activating CD4 cells. The spot size distributions were assessed by morphometric analysis. The assay results were also analyzed by 10 different laboratories.

## Results

The analysis of antigen-elicited ELISPOT sizes for IFN-γ, IL-2, IL-4, IL-5 and IL-17 was found to exhibit a Log-Normal distribution pattern for all 24 donors, and for all CD4 and CD8 cell-derived cytokine signatures. The significance levels were over 5% according to Kolmogorov-Smirnov test. When the spot counts were established in 10 different laboratories using gating criteria established based on Log Normal distributions, the coefficient of variation (CV) of mean spot counts between different laboratories was 6.7%. In contrast, when the participating scientists set gates based on subjective assessment, the CV of mean spot counts obtained between the different laboratories was 26.7%.

## Conclusions

For all 24 donors, 34 antigens, and for all five cytokines studied, ELISPOTs were observed to follow log normal distribution. This statistical function permits us to set upper and lower size gates automatically with a 98% confidence. Using this statistics-based approach, ten different laboratories obtained close to identical counts, as opposed to when the gates were set subjectively by the different investigators. Harmonization of accurate and objective ELISPOT counts can be accomplished based on statistical principles.

